# Nonlinear dynamics and control of a 3-echelon chaotic supply chain system having two stable equilibrium points

**DOI:** 10.1038/s41598-026-46161-1

**Published:** 2026-04-14

**Authors:** Muhamad Deni Johansyah, Sundarapandian Vaidyanathan, Rameshbabu Ramar, Nik Hazimi Mohammed Foziah, Ema Carnia, Chittineni Aruna, Aceng Sambas

**Affiliations:** 1 Department of Mathematic, Universitas Padjadjaran, Jatinangor, 45363 Indonesia; 2 Centre for Control Systems, Vel Tech University, Avadi, Chennai, Tamil Nadu 600062 India; 3 Centre of Excellence for Research, Value Innovation and Entrepreneurship (CERVIE), UCSI University, UCSI Heights, Cheras, Kuala Lumpur, 56000 Malaysia; 4 Department of Electronics and Communication Engineering, V.S.B. Engineering College, Karur, Tamil Nadu 639111 India; 5 Faculty of Business and Management, Universiti Sultan Zainal Abidin, Kuala Nerus, Terengganu 21300 Malaysia; 6 Department of Computer Science and Engineering, KKR & KSR Institute of Technology and Sciences, Guntur, Andhra Pradesh 522017 India; 7 Artificial Intelligence Research Centre for Islam and Sustainability (AIRIS), Universiti Sultan Zainal Abidin, Gongbadak, Kuala Nerus, 21300 Malaysia; 8 Department of Mathematical Sciences, Saveetha School of Engineering, SIMATS, Chennai, Tamilnadu India; 9 Department of Mechanical Engineering, Universitas Muhammadiyah Tasikmalaya, Tasikmalaya, 46196 Indonesia

**Keywords:** Chaotic attractors, Chaos, Supply chain models, Multistability, Offset boosting, Engineering, Mathematics and computing, Physics

## Abstract

This research work introduces a novel 3-echelon chaotic supply chain model incorporating a sinusoidal nonlinearity to represent modeling uncertainty, extending the Anne supply chain model. Through analytical calculations, we show that the new model exhibits chaotic behavior, verified by Lyapunov exponents and Kaplan–Yorke dimension. The largest Lyapunov value of the new model is 1.8336, which is higher than the existing Anne supply chain model. Interestingly, the proposed model has one unstable and two stable equilibrium points, which is rare in the literature. The dynamical analysis is carried out using classical nonlinear tools such as bifurcation plots and Lyapunov exponent spectra. Additionally, the multistability phenomenon is observed in the new model through bifurcation analysis and attractor plots. Finally, 1D and 2D offset boosting control schemes are applied to regulate the position of the attractor without affecting the chaotic dynamics of the system. In future research, these control strategies can be leveraged to optimize resource allocation and waste reduction, thereby enhancing the long-term sustainability of complex supply chain networks.

## Introduction

The chaotic system is a special type of nonlinear dynamical system, characterized by unpredictability and highly sensitivity on initial conditions. Due to this complex dynamics, the chaotic system makes them suitable for many practical applications including robotics^1^, and encryption^2,3^.

The supply chain is crucial to a country’s economy because it connects production, distribution, and consumption in a way that ensures goods and services move efficiently from producers to consumers^4,5^. A well-functioning supply chain reduces operational costs, minimizes delays, and enhances competitiveness in both domestic and international markets^6,7^. It supports industries by ensuring raw materials and components are available when needed, thus maintaining productivity and job stability^8^. In developing economies, efficient supply chains attract foreign investment and boost export capabilities. In advanced economies, they foster innovation, speed, and responsiveness to market demand^9^. Disruptions in the supply chain—such as during a pandemic or geopolitical crisis—can lead to inflation, product shortages, and economic slowdown, showing how deeply integrated and essential supply chains are to national economic health and growth^10^.

Chaos in a supply chain defined as unpredictable and sensitive responses to small changes in system parameters, which can lead to erratic fluctuations in demand, inventory, and production levels^11,12^. This chaotic behavior makes it difficult for supply chain managers to forecast accurately, plan efficiently, or maintain stability, especially in multi-echelon systems where delays and feedback loops are present^13,14^. Even minor variations in order quantity or delivery time can amplify throughout the chain—a phenomenon known as the “bullwhip effect”—resulting in overstocking, stockouts, or increased costs^15–17^. Understanding and modeling chaos in supply chains is crucial, as it enables the identification of critical parameters that trigger instability and supports the development of strategies such as robust control mechanisms or coordination policies to mitigate chaotic effects and improve system resilience^18–20^.

Chaos in supply chains significantly impacts key elements such as safety stock, information distortion, and retailer order satisfaction^21^. Under chaotic conditions, the unpredictable fluctuations in demand and supply lead to inconsistent inventory levels, making it difficult to maintain optimal safety stock—either resulting in overstocking, which increases holding costs, or stockouts, which disrupt service^22,23^. Information distortion, often amplified by delays and nonlinear feedback, causes inaccurate demand signals to be transmitted across the supply chain, further intensifying the bullwhip effect^24^. This leads to poor decision-making at various stages, from production to distribution. As a result, retailer order satisfaction deteriorates, as erratic supply patterns and mismatches between demand and inventory levels prevent timely and accurate fulfillment of customer orders, ultimately weakening customer trust and supply chain efficiency^25,26^.

In recent years, the study of chaotic supply chain models has become a valuable approach to understanding the unpredictable nature of supply chains under certain conditions. Long et al.^27^ explored the dynamic characteristics and resilience issues of multi-echelon supply chain systems against disruptions. It seeks to develop a robust decision-support strategy that ensures the sustainability and resilience of supply chains in volatile markets. The integration of control theory with machine learning techniques, specifically the Echo State Network (ESN) model, enhances the resilience and sustainability of the supply chain against market volatility. Mishra et al.^28^ investigate the interaction between supply chain models and financial models by integrating them through synchronization. Specifically, the research aims to synchronize a fractional-order financial model with a fractional-order supply chain model to capture their chaotic behaviors and interdependencies. They present the significance of integrating financial considerations into supply chain analytics to enhance the robustness and resilience of supply chain operations. Cuong et al.^29^ developed a robust optimization framework for supply chain network design that accounts for uncertainties in parameters such as demand, supply, and transportation costs. The goal is to create a model that ensures optimal performance of the supply chain network under various uncertain scenarios. They suggest that practitioners and decision-makers should consider robust optimization techniques to improve the resilience of supply chain networks in uncertain environments. Sepestanaki et al.^30^ analyzed the chaotic dynamics of a three-echelon supply chain system and developed a control strategy that ensures its stabilization in finite time. They designed a Super-Twisting Sliding Mode (STSM) controller based on adaptive continuous barrier functions to achieve global finite-time stability of the system.

Despite the extensive research on chaotic behavior in multi-echelon supply chain systems (e.g., Anne et al.‘s model), most existing models utilize quadratic nonlinearities only, lacking sufficient representation of modeling uncertainties. Additionally, prior works have not thoroughly explored the implications of symmetry, multistability, and offset-based attractor manipulation in such systems. The absence of these aspects constrains the development of controllable chaotic supply chain models, particularly in environments characterized by high sensitivity and volatility.

The supply chain models discussed on the literature mainly focus on nonlinear dynamics and chaos generation through bifurcation structure and Lyapunov exponent analysis, and its synchronization strategy. Most of the existing models lack to capture the modeling uncertainties using sinusoidal nonlinear term in real world supply chains. Moreover, limited attentions have been given to multidimensional offset boosting and multistability phenomena. In real supply chain environments, nonlinear interactions among production, inventory, and demand can lead to the presence of multiple stable equilibrium points, where the system’s long-term behavior strongly depends on initial conditions.

To address this gap, the paper studies a new chaotic supply chain model incorporate with sinusoidal nonlinearity to capture modeling uncertainty. The present study explores chaos and systematically verified various complex dynamic behaviors such as stable equilibrium points, multistability and 2D offset boosting control techniques. Based on the motivation above, we have introduced a novel 3-echelon chaotic supply chain model that integrates a sinusoidal nonlinearity to explicitly account for modeling uncertainty. This extension of the Anne model is analyzed through bifurcation diagrams, Lyapunov exponents, and offset boosting control techniques. The proposed system reveals more complex dynamics, including chaos, multistability, and rotation symmetry, and allows for customized control of attractor positions via 2D offset boosting, without altering the underlying chaotic nature.

The rest of this paper is organized as follows: Sect. 2 introduces the proposed supply chain model; Sect. 3 discusses the dynamic analysis including bifurcation and Lyapunov spectra; Sect. 4 investigates multistability; Sect. 5 presents offset boosting control; and Sect. 6 concludes with key findings and future directions.

## New 3-echelon chaotic supply chain model

Supply chain models have been studied by many researchers in the literature. A 3- echelon supply chain model consists of three decision making levels such as retailer, wholesaler and manufacturer. In compared with other supply chain model, 3 echelon models is distinguished by higher dimensionality and stronger inter-echelon coupling, which make it more realistic and capable of exhibiting richer dynamic behaviours.

A popular supply chain model is a 3-echelon chaotic supply chain model prescribed by Anne et al.^31^. Anne chaotic supply chain model is a nonlinear dynamical system with two quadratic nonlinearities. It is a generalized form of the famous Lorenz chaotic system^32^. Anne et al.^31^ introduced a nonlinear 3-echelon supply chain model that covers safety stock, information distortion, and retailer order satisfaction. Anne nonlinear supply chain model was defined in^31^ as follows:

1 $$\left\{ \begin{gathered} {{\dot {y}}_1}=m{y_2} - (n+1){y_1} \hfill \\ {{\dot {y}}_2}=r{y_1} - {y_2} - {y_1}{y_3} \hfill \\ {{\dot {y}}_3}={y_1}{y_2} - (k+1){y_3} \hfill \\ \end{gathered} \right.$$

where $\:{y}_{1}$ is the load demanded by the retailer in the current period, $\:{y}_{2}$ represents the load distributors can supply in the current period, and $\:{y}_{3}$ represents the load produced in the current period depending on the order. The economic interpretation of the constants $\:m,n,r,k\:$can be explained as follows: $\:m$ denotes the rate of demand satisfaction with the retailer, $\:n\:$denotes the inventory level of the distributors in the market, $\:k$ represents the safety stock coefficient of the manufacturer, and $\:r$ is the rate of information distortion of the commercial products demanded by the retailer. As noted in^31^, if $\:m=n+1$ and $\:c=k+1,$ then the system (1) reduces to the Lorenz 3-D system^32^ as given below:

2 $$\left\{ \begin{gathered} {{\dot {y}}_1}=m({y_2} - {y_1}) \hfill \\ {{\dot {y}}_2}=r{y_1} - {y_2} - {y_1}{y_3} \hfill \\ {{\dot {y}}_3}={y_1}{y_2} - c{y_3} \hfill \\ \end{gathered} \right.$$

This simple calculation shows that the model (1) is a generalized form of the famous Lorenz chaotic system (2). The model (1) was studied in detail by Wang et al.^33^, who observed chaotic behavior in the Anne for the parameter values as given below:

3 $$\:m=12,\:\:\:n=7,\:\:r=45,\:\:\:k=\frac{7}{3}\:$$

For $(m,n,r,k)=\left( {12,7,45,7/3} \right)$and $\:Y\left(0\right)=\left(\mathrm{0.3,0.2,0.3}\right),$ the Lyapunov exponents (LyE) for the system (1) were computed for $\:T=1\times\:{10}^{4}$ seconds as follows:

4 $${\mathrm{LyE}}_{{\mathrm{1}}} = {\mathrm{1}}.{\mathrm{121}}0,{\text{ LyE}}_{{\mathrm{2}}} = 0,{\text{ LyE}}_{{\mathrm{3}}} = - {\mathrm{13}}.{\mathrm{4542}}$$

Thus, the model (1) is chaotic and dissipative with the maximal LyE given by LyE_max_ = 1.1210 The Kaplan dimension for the model (1) is found as follows:

5 $$D_{K} = 2 + \frac{{LyE_{1} + LyE_{2} }}{{\left| {LyE_{3} } \right|}} = 2 + \frac{{1.1210 + 0}}{{13.4542}} = 2.0833.$$

In this research paper, we propose a new 3-echelon chaotic supply chain system by modifying the existing model (6) with the introduction of a sinusoidal nonlinearity, which represents a modelling uncertainty and by considering different values of the system parameters. The sinusoidal nonlinear term represents the periodic fluctuations and modelling uncertainty that may happen in real world supply chains such as seasonal demand variations, cyclical market behavior, or periodic policy adjustments.

Thus, we propose a new 3-echelon supply chain system with the following dynamics:

6 $$\left\{ \begin{gathered} {{\dot {y}}_1}=m{y_2} - (n+1){y_1} \hfill \\ {{\dot {y}}_2}=r{y_1}+p\sin ({y_1}) - {y_2} - {y_1}{y_3} \hfill \\ {{\dot {y}}_3}={y_1}{y_2} - (k+1){y_3} \hfill \\ \end{gathered} \right.$$

The term $p\sin ({y_1})$ is introduced into the ${\dot {y}_2}$equation to model a bounded, non-linear perturbation. Mathematically, ${\dot {y}_2}$ represents the rate of change of the second echelon. By making this change dependent on${y_1}$, we introduce a non-linear coupling that more accurately reflects the feedback loops common in chaotic systems. The sinusoidal function ensures that the uncertainty remains within a bounded range $p,$ preventing the model from diverging to infinity while still allowing for complex, chaotic oscillations. From an economic perspective, ${y_2}$ is often sensitive to the fluctuations of ${y_1}$ due to the information distortion. The sinusoidal uncertainty $p\sin ({y_1})$ represents seasonal demand cycles or periodic market fluctuations that are not perfectly predictable. By adding this specifically to ${\dot {y}_2},$we model how the “middle” echelon must adjust its production or orders based on the non-linear signals received from the first echelon, capturing the inherent modeling uncertainty caused by human behavior or external market shocks.

The states and the bifurcation constants in the new supply chain model (6) have the same economical interpretation as the model (1). We claim that the new 3-D supply chain model (6) is chaotic for the parameter values.

7 $$\:m=14,\:\:\:n=11,\:\:r=55,\:\:\:k=6,\:p=0.2\:\:$$

The parameter values are chosen through numerical simulation to explore complex nonlinear dynamics such as chaos and bifurcation in the proposed supply chain model. For $\:\left(m,\:n,\:r,\:k,p\right)=\left(\mathrm{14,11,55,6},0.2\right)$and $\:Y\left(0\right)=\left(\mathrm{0.3,0.2,0.3}\right),$ the LyE values for the new supply chain model (6) were computed using standard Wolf algorithm^34^, which estimates the exponents by monitoring the exponential divergence of nearby trajectories in phase space for $\:T=1\times\:{10}^{4}$ seconds as follows:

8 $${\mathrm{LyE}}_{{\mathrm{1}}} = {\mathrm{1}}.{\mathrm{8336}},{\text{ LyE}}_{{\mathrm{2}}} = 0,{\text{ LyE}}_{{\mathrm{3}}} = - {\mathrm{21}}.{\mathrm{8336}}$$

Thus, the proposed 3-echelon supply chain model (6) is chaotic and dissipative with the maximal LyE given by $Ly{E_{\hbox{max} }}=1.8336.$ The Kaplan dimension (KD) for the proposed supply chain model (1) is found as follows:

9 $${D_K}=2+\frac{{Ly{E_1}+Ly{E_2}}}{{\left| {Ly{E_3}} \right|}}=2+\frac{{1.8336+0}}{{21.8336}}=2.0840.$$

Table 1 gives a comparison of the maximal LyE and the Kaplan dimension (KD) for the Anne 3-echelon supply chain model (1) and the proposed supply chain model (6).

**Table 1:** MLE and KD for the two chaotic supply chain models.

Supply chain system	LyE_max_	KD
Anne system^28^	1.1210	2.0833
Proposed system	1.8336	2.0840

**Fig. 1:**
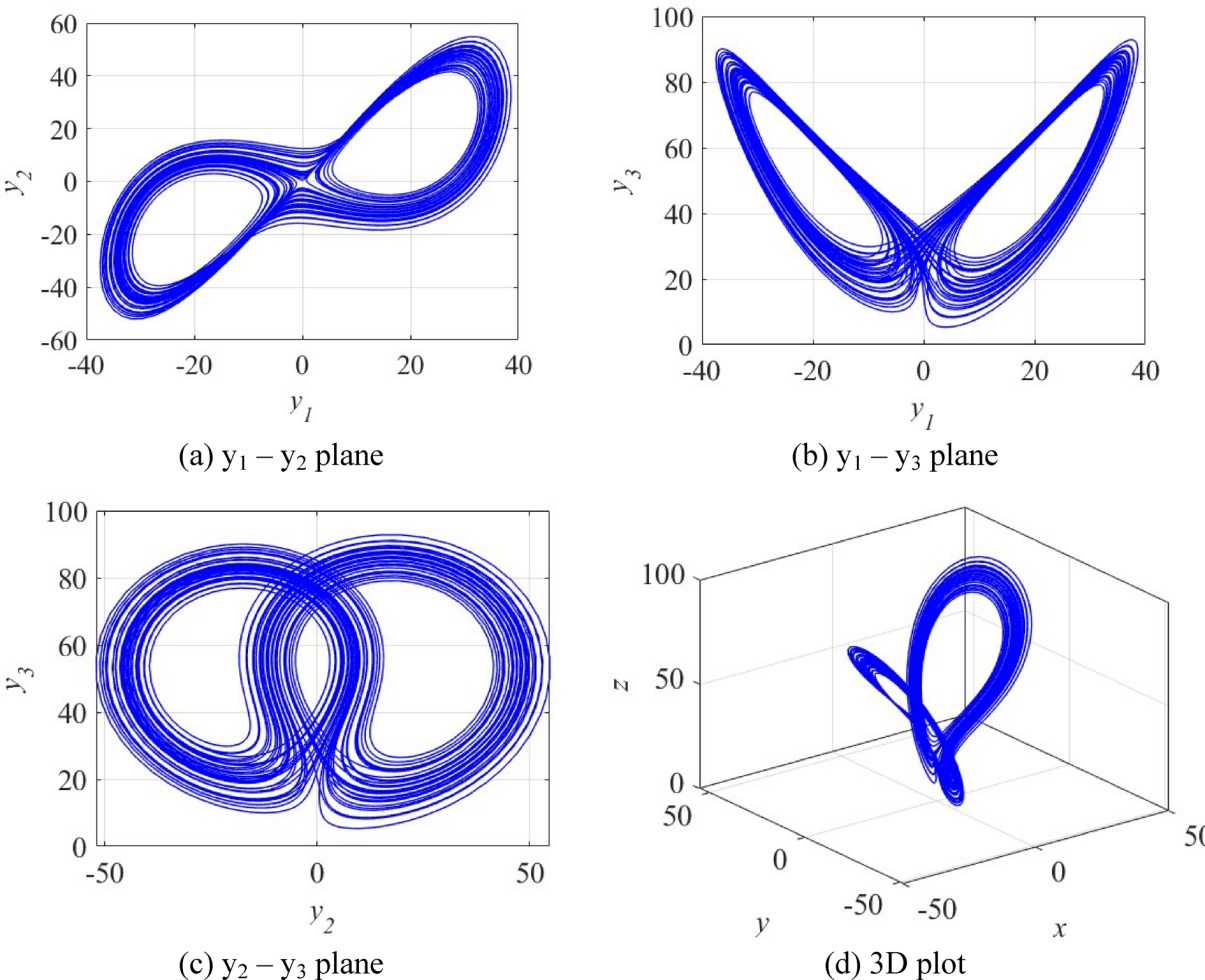
MATLAB simulation plots for the new chaotic supply chain model (6) for the parameter values (*m*, *n*,*r*,* k*,*p*) = (14,11,55,6,0.2) and initial condition (0.3, 0.2, 0.3).

The chaotic supply chain system (6) remains invariant under the coordinates transformation

10 $$({y_1},{y_2},{y_3}) \mapsto ( - {y_1}, - {y_2},{y_3})$$

which shows that the proposed supply chain system (6) has a rotation symmetry about the $\:{y}_{3}$ coordinate axis. The stationary points or the equilibria of the new chaotic supply chain system (6) are got by means of solving the nonlinear system of equations given below:

11a $$my_{2} - (n + 1)y_{1} = 0$$

11b $$ry_{1} + p\sin (y_{1} ) - y_{2} - y_{1} y_{3} = 0$$

11c $$y_{1} y_{2} - (k + 1)y_{3} = 0$$

We solve the system of equations (11) for $\:\left(m,\:n,\:r,\:k,p\right)=\left(\mathrm{14,11,55,6},0.2\right),\:$the parameter values in the chaotic case (7). Equation (11) indeed contains transcendental nonlinear terms, precluding closed-form solutions for the equilibrium points. Accordingly, we employed the standard *fsolve* function in MATLAB, which is a robust numerical root-finding algorithm, for their computation, using multiple initial guesses to identify all physically relevant equilibria. The equilibrium points of the proposed model (6) are given as.

12 $$S_{0} = \left[ {\begin{array}{*{20}c} 0 \\ 0 \\ 0 \\ \end{array} } \right],\;S_{1} = \left[ {\begin{array}{*{20}c} {21.0293} \\ {18.0251} \\ {54.1507} \\ \end{array} } \right],\;S_{2} = \left[ {\begin{array}{*{20}c} { - 21.0293} \\ { - 18.0251} \\ {54.1507} \\ \end{array} } \right].$$

The Jacobian matrix of the system (6) can be written as

13 $$J = \left| {\begin{array}{*{20}c} { - (n + 1)} & m & 0 \\ {r - y_{3} + p\cos y_{1} } & { - 1} & { - y_{1} } \\ {y_{2} } & {y_{1} } & { - (k + 1)} \\ \end{array} } \right|$$

At S0 (0,0,0) and (m, n, r, k, p) = (14,11,55,6,0.2), the matrix (13) becomes as

14 $$J = \left| {\begin{array}{*{20}c} { - 12} & {14} & 0 \\ {55.2} & { - 1} & 0 \\ 0 & 0 & { - 7} \\ \end{array} } \right|$$

The eigenvalues of the Jacobian matrix (14) of the new chaotic supply chain system (6) at $\:{S}_{0}$ are determined as

15 $$({\lambda _1},{\lambda _2},{\lambda _3})=(~ - 7, - 34.8381,21.8381)$$

Equation (15) shows that the stationary point $\:{S}_{0}$ is an unstable, saddle point equilibrium of the system (6).

At S_1_ (21.0293, 18.0251, 54.1507) and $\:\left(m,\:n,\:r,\:k,p\right)=\left(\mathrm{14,11,55,6},0.2\right)$, the matrix (13) becomes as

16 $$J=\left| {\begin{array}{*{20}{c}} { - 12}&{14}&0 \\ {1.036}&{ - 1}&{ - 21.0293} \\ {18.0251}&{21.0293}&{ - 7} \end{array}} \right|$$

The eigenvalues of the Jacobian matrix (16) of the new chaotic supply chain system (6) at $\:{S}_{1}$ are determined as

17 $$({\lambda _1},{\lambda _2},{\lambda _3})=(~ - 19.9801, - 0.0096 \pm j23.0289)$$

Equation (17) shows that the stationary point $\:{S}_{1}$ is a stable equilibrium point, characterized by negative eigenvalues.

Due to the rotation symmetry of the supply chain system (6) about the $\:{y}_{3}-$coordinate axis, the eigenvalues of the Jacobian matrix of the new chaotic supply chain system (6) at $\:{S}_{2}$ coincide with the eigenvalues of the Jacobian matrix of the new system (6) at $\:{S}_{1}.$ Hene, the stationary point $\:{S}_{2}$ is also a stable equilibrium point.

Figure 1 give the MATLAB (version R2024b) plots of the new chaotic supply chain system (6) for the parameter values $\:\left(m,\:n,\:r,\:k,p\right)=\left(\mathrm{14,11,55,6},0.2\right),$ and $\:Y\left(0\right)=\left(\mathrm{0.3,0.2,0.3}\right).$.

## Analysis of system dynamics

In this section, the dynamical analyses of the new supply chain model (6) were performed through bifurcation analysis and LyE spectrum. A bifurcation diagram provides the visual representation of how the states of the system transition between periodic and chaotic as the system parameter vary. The diagram reveals stable/unstable fixed points, emergence of oscillations and transition to chaotic behaviour. The LyE spectrum gives the quantitative measure of the sensitivity on initial conditions, as essential behaviour of chaotic systems. In this work, all the numerical simulations are carried out using fourth order Runge – Kutta method with the step size of h = 0.01, and the initial conditions (0.3, 0.2, 0.3). The bifurcation diagrams are plotted based on local maxima of the state variable *y*_1_.

**Fig. 2:**
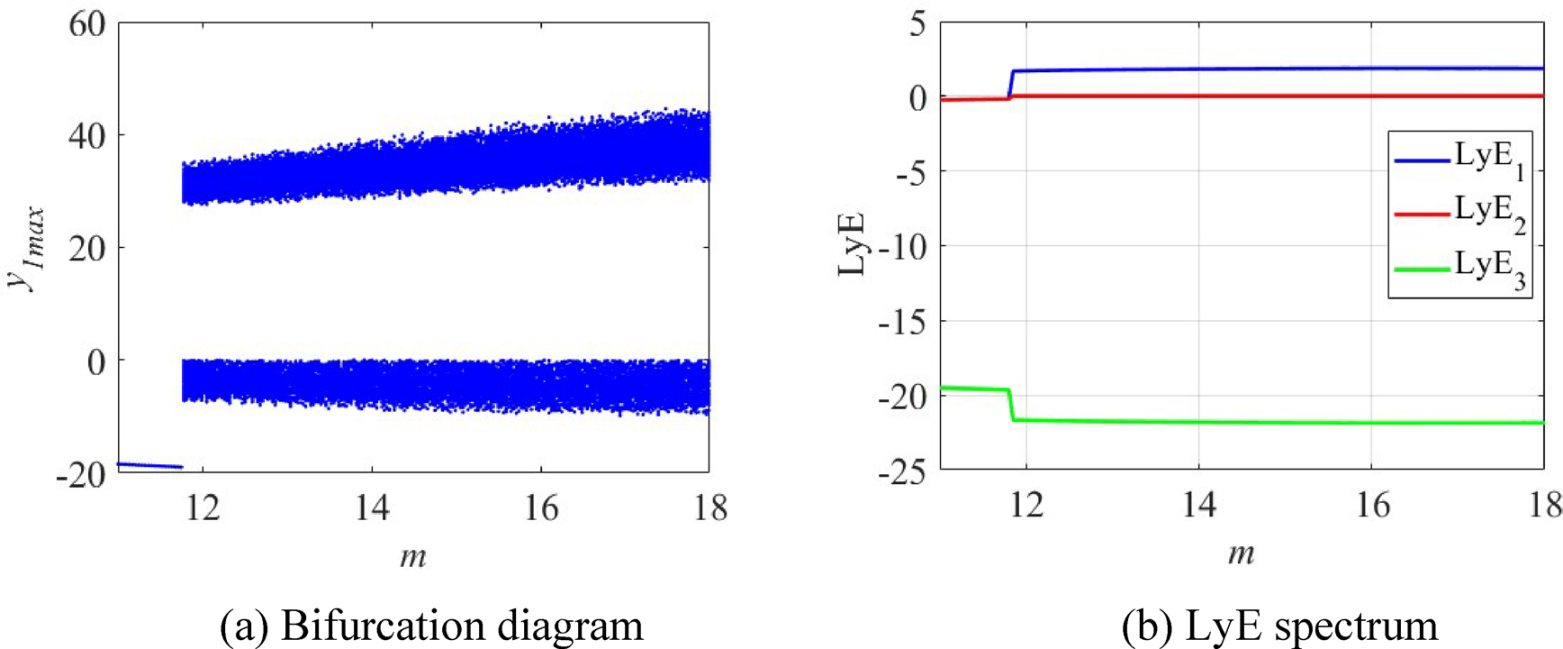
(**a**) Bifurcation diagram (**b**) LyE spectrum for the parameter $$m \in [11,18],\left( {n,~r,~k,p} \right)=$$$$\left( {11,55,6,0.2} \right)$$.

**Fig. 3:**
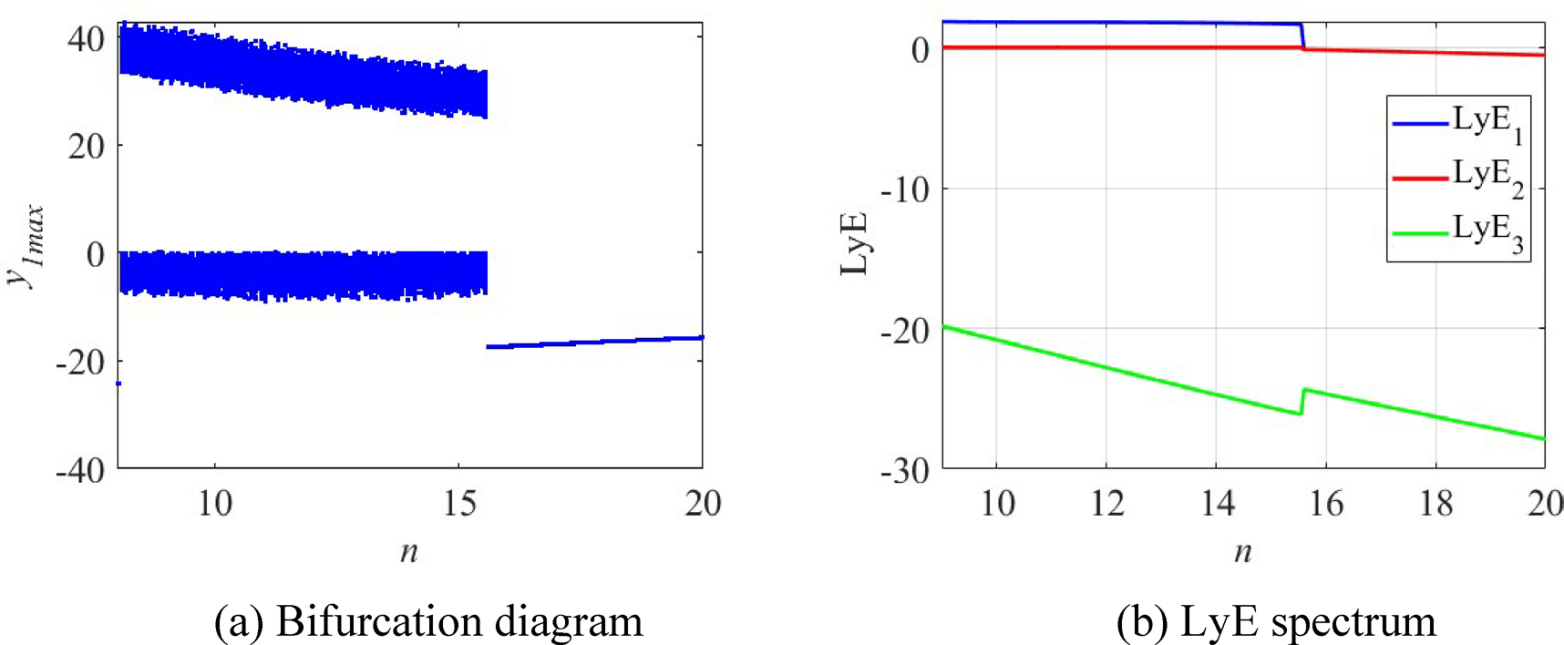
(**a**) Bifurcation diagram (**b**) LyE spectrum for the parameter $$n \in [9,20],\left( {m,~r,~k,p} \right)=$$$$\left( {14,55,6,0.2} \right)$$.

To confirm the presence of chaotic oscillations in the proposed model (6), we plotted bifurcation diagram and LyE spectrum using the initial condition *Y(*0) = (0.3 0.2 0.3), where LyE spectrum distinguishes LyE_1_ in blue, LyE_2_ in red and LyE_3_. In green. First, we plotted for the parameter *m* as given in Fig. 2. In Fig. 2a, the single line with in $11<m<12$indicates that the system settles to a steady value and then the diagram turns in to dense band for $m>12$ represents the chaotic behavior in the proposed model. It is also clearly understand from Fig. 2b that the system exhibits periodic behavior with in $11<m<12$ where LyE_1_ = LyE_2_ = 0 and LyE_3_ < 0 and chaotic oscillation for $m>12$, where LyE_1_ > 0, LyE_2_ = 0 and LyE_3_ < 0.

The bifurcation diagram and LyE spectrum for the system parameter *n* are shown in Fig. 3. Figure 3a reveals the chaos through the double dense band within the region $9<n<15.8$ and sensitive depends on initial conditions in the new system. While the single line ($n>15.8$) signifies convergence to a steady state and the stable equilibrium. Similarly, Fig. 3b also further confirms the chaos via positive LyE values in the region $9<n<15.8$ and periodic dynamics where LyE_1_ = LyE_2_ = 0 and LyE_3_ < 0.

**Fig. 4:**
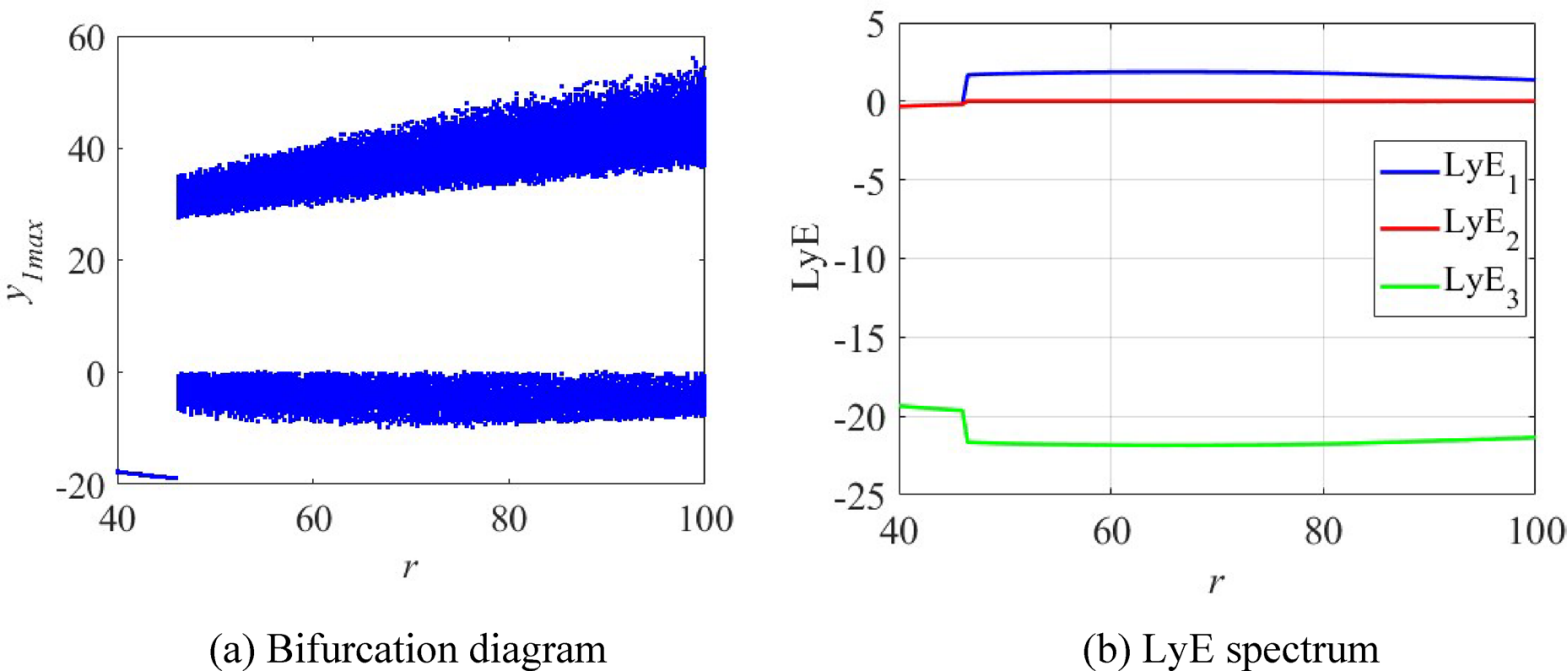
(**a**) Bifurcation diagram (**b**) LyE spectrum for the parameter $$r \in [40,100],\left( {m,n,~k,p} \right)=$$$$\left( {14,11,6,0.2} \right)$$.

**Fig. 5:**
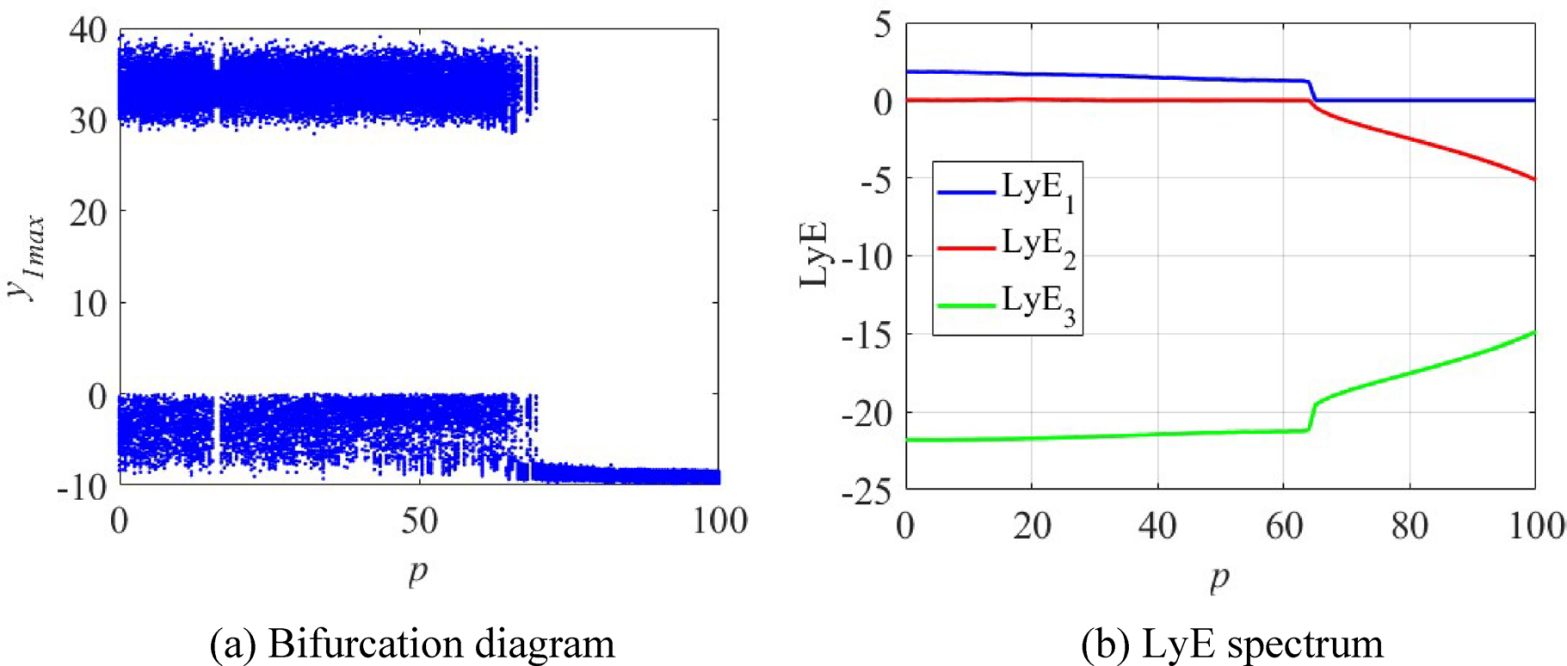
(**a**) Bifurcation diagram (**b**) LyE spectrum for the parameter $$p \in [0,100],\left( {m,n,~r,k} \right)=$$$$\left( {14,11,55,6} \right)$$.

Figure 4 shows the bifurcation diagram and LyE spectrum for the parameter variation *r* in the range$40<r<100$. Figure 4a illustrates the bifurcation diagram, where a dense band appears over a prolonged interval$45<r<100$, indicates the onset chaotic dynamics in the system. This is further conformed by the positive LyE values shown in corresponding LyE spectrum in Fig. 4b.

Figure 5a represents the bifurcation diagram of the system (6) with respect to the parameter *p*, where the dense band of scattered points in the region 0 < *p* < 62 indicates the chaotic behavior and highly sensitive to initial conditions, followed by a transition to periodic dynamics as *p* increases further. This is further conformed by LyE spectrum as shown in Fig. 5b, where positive LyE_1_ in the range 0 < *p* < 62 indicate chaotic oscillations, while the transition to periodic behavior is marked by LyE_1_ = 0 with LyE_2_< 0 and LyE_3_ < 0.

**Fig. 6:**
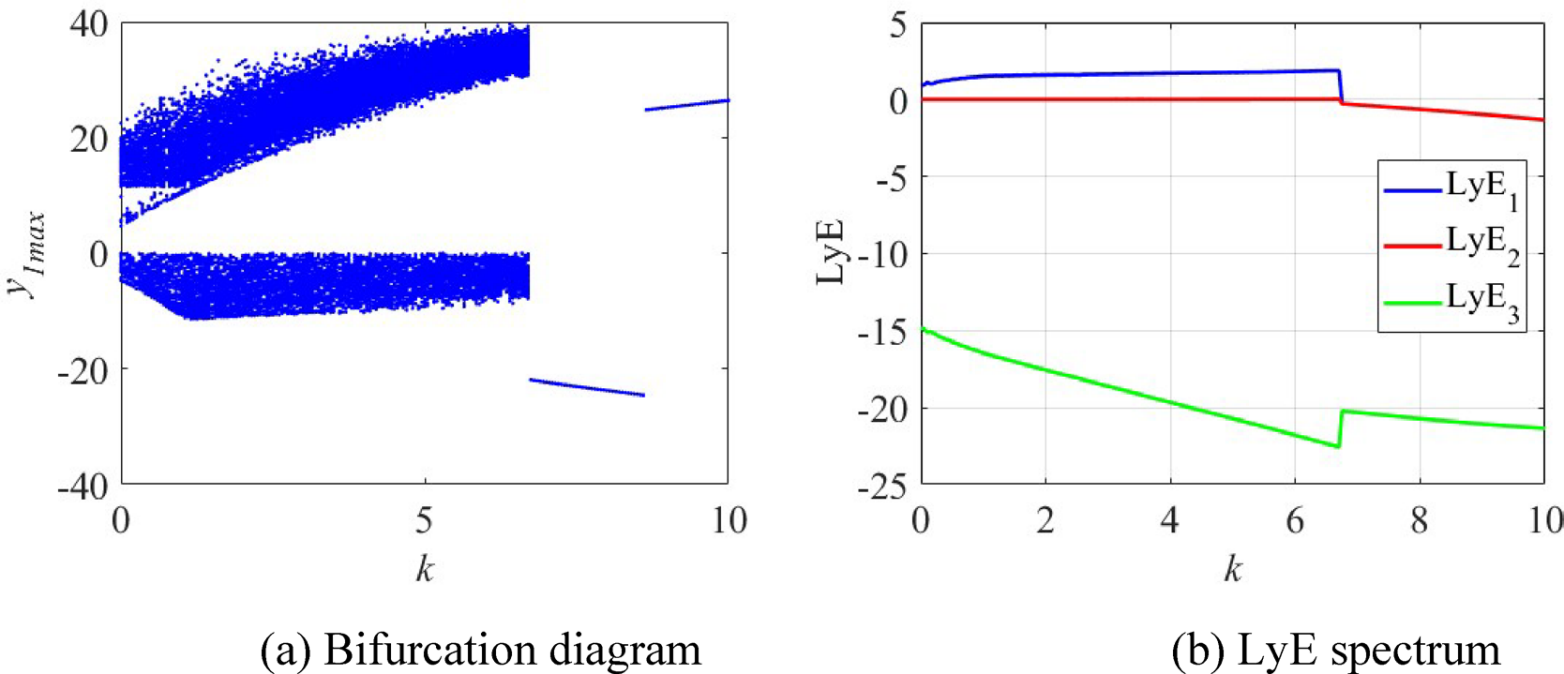
(**a**) Bifurcation diagram (**b**) LyE spectrum for the parameter $$k \in [0,10],\left( {m,n,~p,r} \right)=$$$$\left( {14,11,0.2,55} \right)$$.

Figure 6a represents the bifurcation diagram of the system (6) with respect to the parameter *k*, where the dense band in the region 0 < *k* < 6.5 indicates the chaotic behavior, followed by a transition to stable points as *k* increases further. This is further conformed by LyE spectrum as shown in Fig. 6b, where positive LyE_1_ in the range 0 < *k* < 6.4 indicate chaotic oscillations, while the transition to stable points is marked by negative LyE values.

## Multistability analysis

Multistability phenomenon in a chaotic system refers to the coexistence of attractors under the fixed set of parameters with distinct initial conditions^35–39^. It means that the particular system can settle into different chaotic or periodic oscillation depending on the initial conditions. Multistability in a chaotic system can be demonstrated by plotting the bifurcation diagram for distinct initial conditions^40,41^. When the multiple branches emerge for the same parameter value, resulting from distinct initial conditions confirm the presence of multistability in the system. In a chaotic supply chain model, multistability represents the different operational regimes such as balanced demand and supply shortages, and the final system behaviour strongly depends on initial inventory levels, order rates or demand conditions. This helps us to understand why the similar supply chains present markedly different performance outcomes despite having comparable structural settings.

**Fig. 7:**
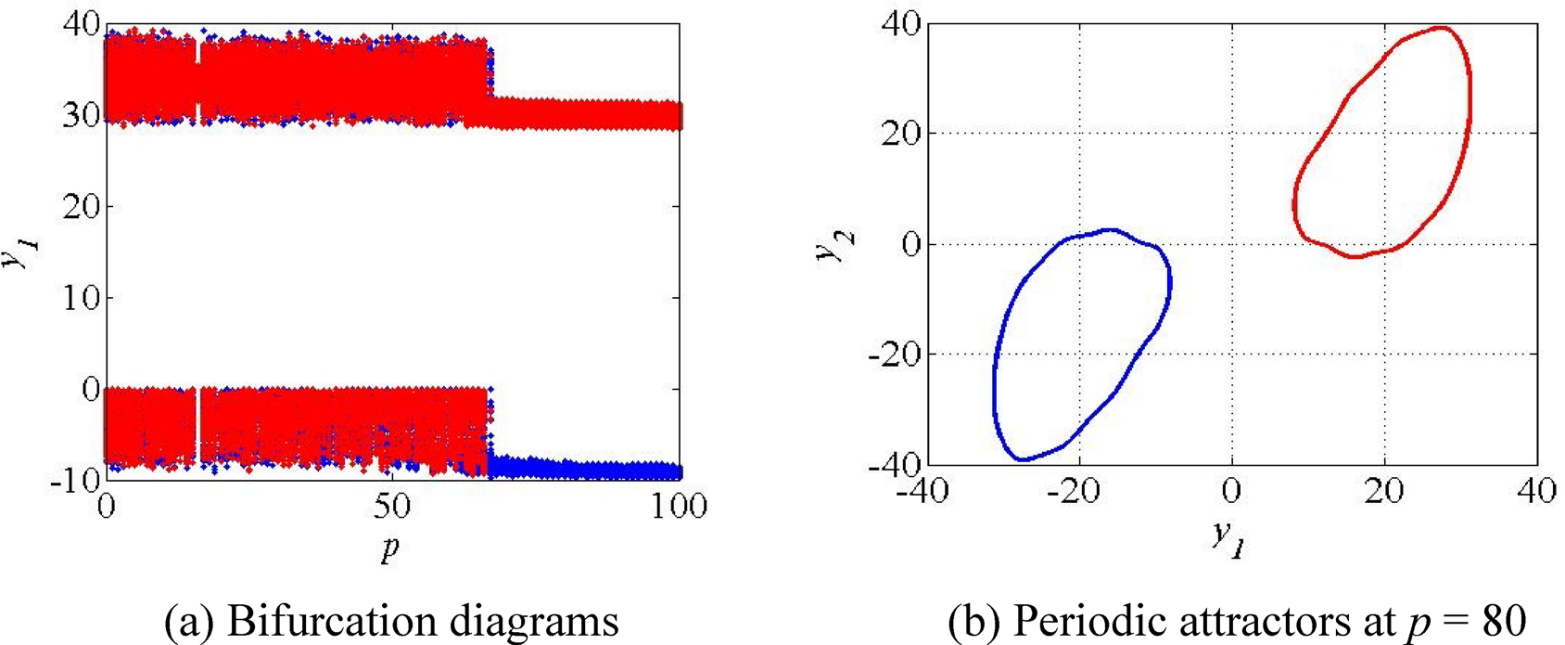
(**a**) Bifurcation diagrams (**b**) Coexisting periodic attractors of the 3D-echelon chaotic supply chain model (6) with the initial conditions *Y*_1_(0) = (0.3, 0.2, 0.3), shown with blue branches, and *Y*_2_(0) = (-0.3, 0.2, -0.3), shown with red branches.

Figure 7a shows the bifurcation diagram of the system (6) as the function of system parameter *p*. The diagram includes two set of initial conditions *Y*_1_(0) = (0.3, 0.2, 0.3), shown with blue branches, and *Y*_2_(0) = (-0.3, 0.2, -0.3), shown with red branches. For parameter values beyond *p* = 62, two distinct branches appear for the same values of *p*. This behavior indicates the presence of multistablity in the system. It also suggests the existence of coexisting attractors. Figure 7b provides clear evidence of coexisting periodic attractors at *p* = 80.

## Offset boosting control

DC offset boosting control in a chaotic system refers to a control technique aimed to relocate the attractors of a chaotic system by adding a constant (DC) offset to variables of the system^42,43^. 2D offset boosting is an advanced form of DC offset control in which constant values are added to two of the system’s state equations.

In the proposed model, the two variables${y_2}$and${y_3}$receive constant additive offsets as represented in (18), where$\alpha$and$\beta$are constant DC offsets. The third variable${y_1}$remains unaltered. The parameters$\alpha$and$\beta$relocates the phase portraits along${y_2}$and${y_3}$ while without editing the other dynamics of the proposed system. When the control parameters are positive, the offset moves in the negative direction of the state variables and negative parameters move in the opposite direction. The added control parameters also convert the unipolar to bipolar signal or vice versa.

18 $$\left\{ \begin{gathered} {{\dot {y}}_1}=m({y_2}+\alpha ) - (n+1){y_1} \hfill \\ {{\dot {y}}_2}=r{y_1}+p\sin ({y_1}) - ({y_2}+\alpha ) - {y_1}({y_3}+\beta ) \hfill \\ {{\dot {y}}_3}={y_1}({y_2}+\alpha ) - (k+1)({y_3}+\beta ) \hfill \\ \end{gathered} \right.$$

**Fig. 8:**
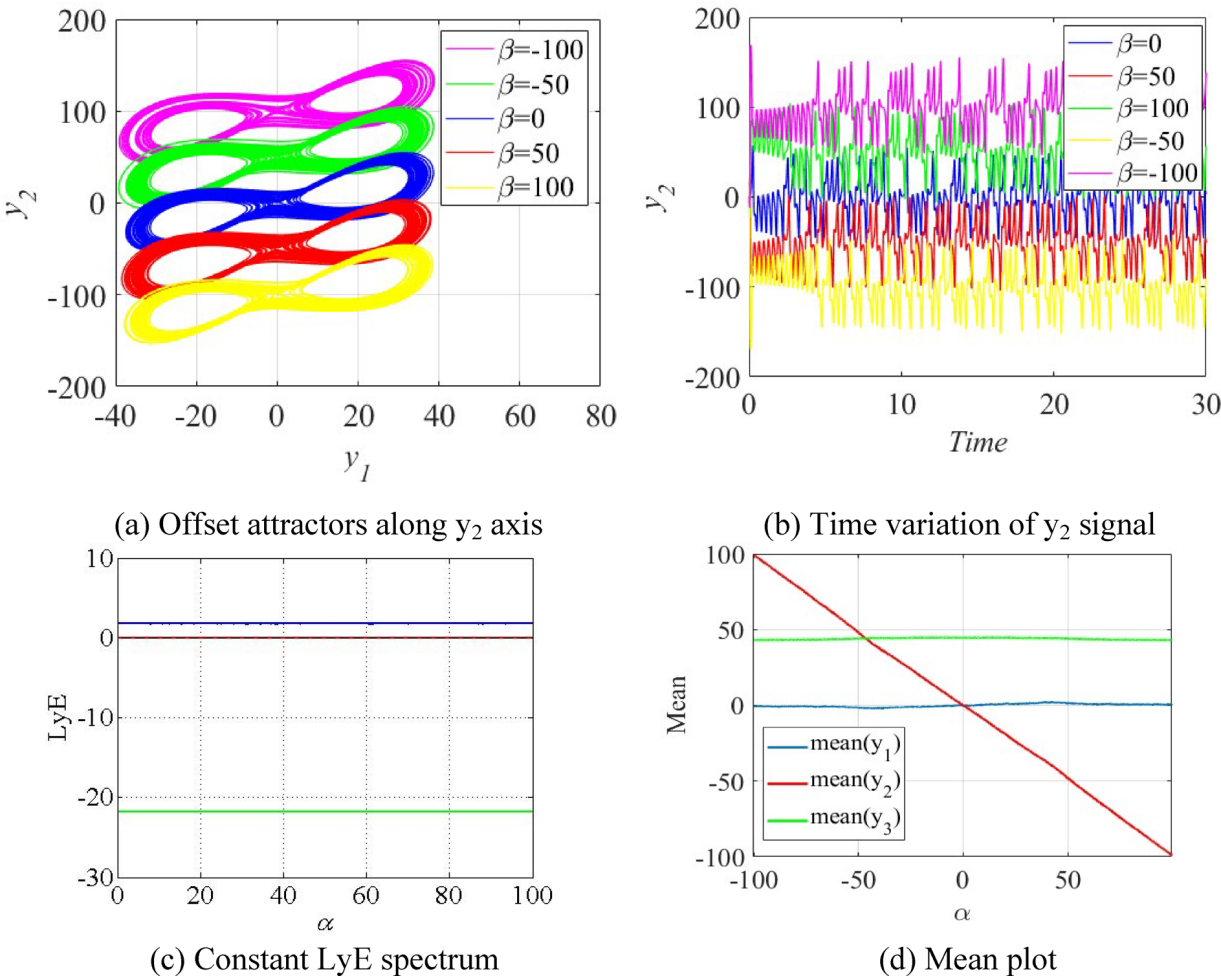
1D offset boosting when $\alpha \ne 0,\beta =0$ (**a**) Coexisting attractors in *y*_1_ – *y*_2_ plane; (**b**) Time variation of y_2_ signal (**c**) LyE spectrum as the function of $\alpha$. (**d**) Mean values of state signals as a function of $\alpha$.

Case 1: When $\alpha \ne 0,\beta =0$, the phase portraits of the system (12) are relocated along only *y*_2_ plane as shown in Fig. 8a, in which$\alpha =0$(Blue), $\alpha =50$(Red), $\alpha = - 50$(Green), $\alpha =100$(Yellow) and $\alpha = - 100$(Magenta). The corresponding time variation of *y*_2_ signal is shown in Fig. 8b in which the bipolar is converted in to an unipolar signal. The invariant LyE spectrum as a function of$\alpha$is shown in Fig. 8c which indicates that the added control parameter $\alpha$ does not modify the LyE values and chaotic behavior of the proposed model. If the state variable *y*_2_ is modified by adding the constant$\alpha$, this affects the average of y_2_ as shown in Fig. 8d, while the average of other variables remains unchanged.

In the proposed model, the state variable *y*_2_ represent the variations of load distributors from their nominal operating points. Therefore, negative values in variable *y*_2_​ should be interpreted as deviations below the reference level rather than physically negative inventory. In practical supply chain terms, such negative deviations may correspond to situations like temporary backorders, inventory shortages, or corrective adjustments in order quantities.

**Fig. 9:**
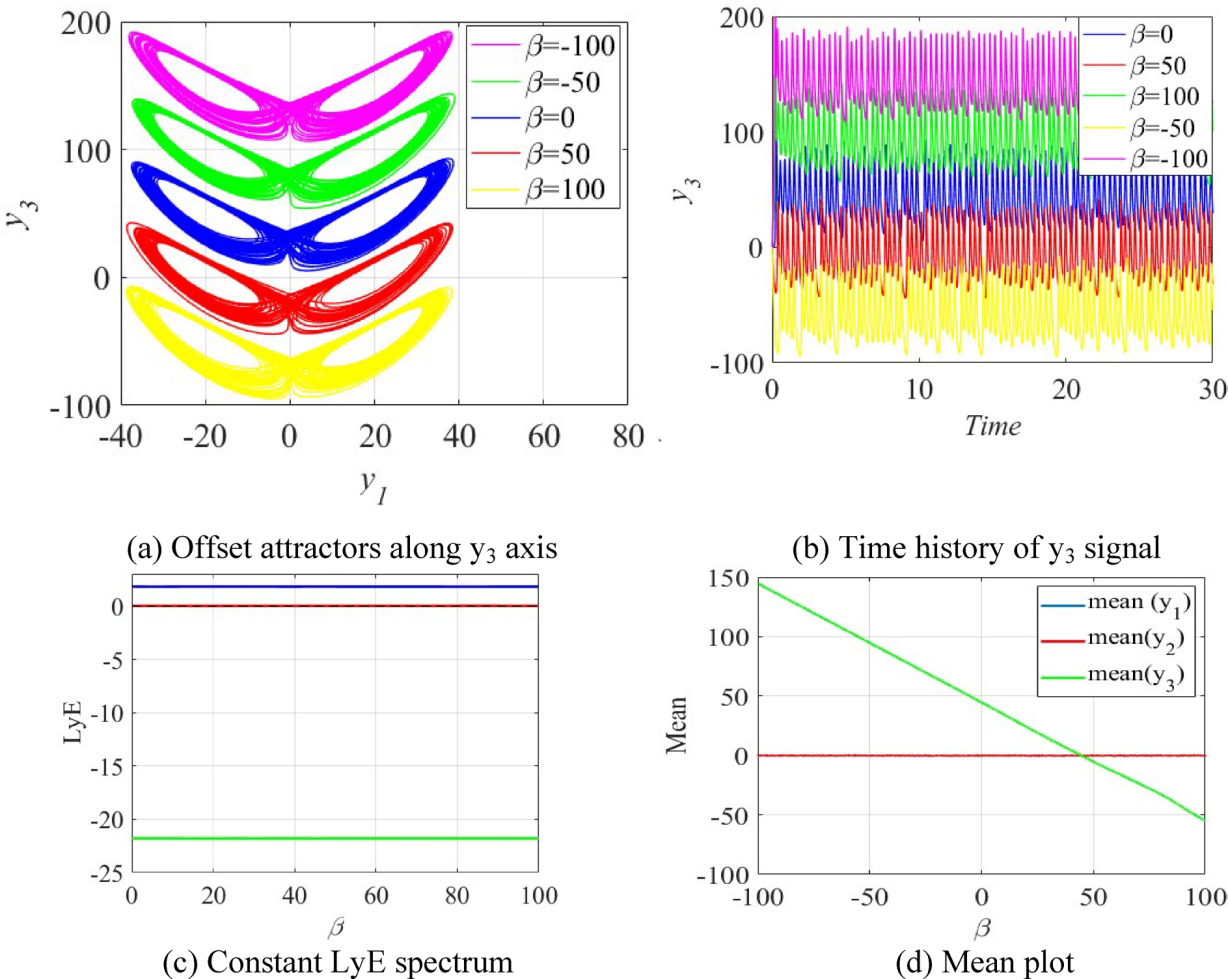
1D offset boosting when$\beta \ne 0,\alpha =0$ (**a**) Coexisting attractors in *y*_1_ – *y*_3_ plane (**b**) Time variation of y_3_ signal (**c**) LyE spectrum as the function of $\beta$ (**d**) Mean values of state signals, where mean($\:{y}_{1}$) = mean ($\:{y}_{2}$).

Case 2: When$\beta \ne 0,\alpha =0$, the phase portraits of the system (12) are shifted along only *y*_3_ plane as shown in Fig. 9a, in which$\beta =0$(Blue), $\beta =50$(Red), $\beta = - 50$(Green), $\beta =100$(Yellow) and $\beta = - 100$(Magenta). The corresponding time variation of *y*_3_ signal is shown in Fig. 9b. The invariant LyE spectrum as a function of$\beta$is shown in Fig. 9c which indicates that the inclusion of control parameter$\beta$ also does not modify the LyE values and chaotic behavior of the proposed model. Since the state variable *y*_3_ is modified by the parameter$\beta$, the average of only y_3_ is changed according$\beta$ value as shown in Fig. 9d, while the average of other variables remains unchanged. The mean ($\:{y}_{1}$) is not visually distinct in Fig. 8(b) because it is perfectly superimposed by mean ($\:{y}_{2}$).

**Fig. 10:**
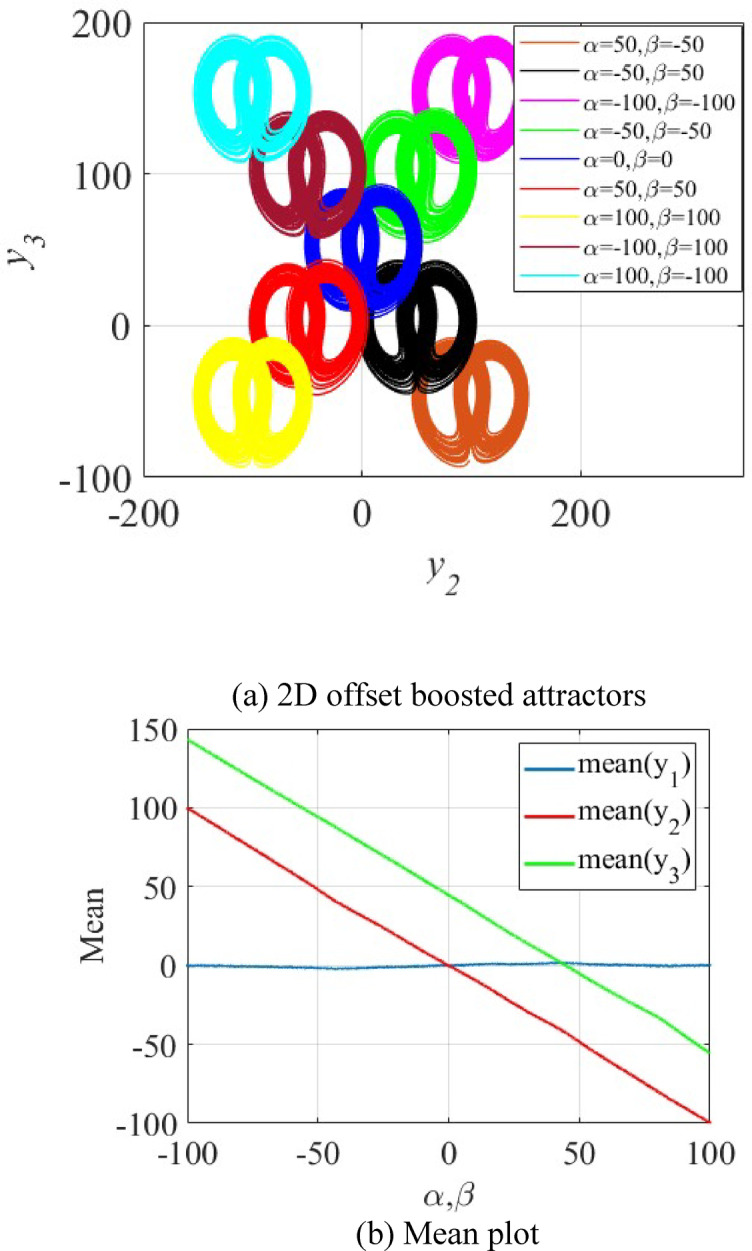
2D offset boosting when$\alpha \ne 0,\beta \ne 0$ (**a**) Coexisting attractors in *y*_2_ – *y*_3_ plane (**b**) Mean values of state signals as a function of$\alpha$and$\beta$.

Case 3: When$\alpha \ne 0,\beta \ne 0$, the phase portraits of the system (12) are shifted along both *y*_2_ and *y*_3_ plane as shown in Fig. 10a. In this case, the average of both *y*_2_ and *y*_3_ states is modified according to the booster parameter $\alpha$and $\beta$ values as shown in Fig. 10b.

Without changing the inherent nonlinear dynamics, offset boosting allows for the simultaneous and independent shifting of several state variables in a chaotic supply chain system, such as supply and production levels. This makes it possible for various supply chain tiers to coordinately modify their baseline operating points, guaranteeing alignment between production output and distribution capacity in the face of shifting market conditions. Multi-dimensional offset boosting maintains the adaptive and responsive properties of chaotic dynamics while improving operational flexibility, resilience, and robustness against demand fluctuations and disruptions by moving the system’s attractor to more desirable regions of the state space.

The offset boosting is used to regulate the undesirable chaotic oscillations in demands, inventory, and production dynamics. In real-world applications, this control mechanism represents the adjustment of production rates and regulating inventory replenishment policies. The implementation of these strategies may cause additional operational costs. Therefore, from a cost–benefit perspective, the implementation of such control strategies can lead to improved operational efficiency, reduced holding and shortage costs, and more stable supply chain performance, which may outweigh the implementation costs in many practical scenarios.

## Limitations and future work

The proposed model has several limitations. The sinusoidal nonlinearity represents the continuous fluctuation and modelling uncertainty in the interaction between demand, inventory, and production levels. The introduction of sinusoidal nonlinearity helps us to explore chaos, multistability and attractor’s position control in supply chain models. However, it does not capture the abrupt disturbances that frequently occur in the real world supply chains. The model is constructed using fixed system parameters and deterministic demands; whereas the practical supply chains are influenced by stochastic demand fluctuations, parameter uncertainties, and random disruptions. Future work may involve the application of fractional-order calculus, real-time data integration, incorporating stochastic disturbances, and adaptive control strategies to further enhance the model’s relevance and applicability to complex, real-world supply networks.

## Conclusion

In this paper, we introduced a new 3-echelon chaotic supply chain model that incorporates a sinusoidal nonlinearity to account for modeling uncertainty, extending the classical Anne model. Our findings reveal that the proposed model not only exhibits chaotic dynamics with a butterfly-shaped attractor but also demonstrates unique features such as multistability, rotational symmetry, and controllable attractor displacement via 2D offset boosting techniques. Through a detailed bifurcation analysis and Lyapunov exponent computations, we identified critical parameters influencing the system’s transition from stable to chaotic behavior. The model shows sensitivity to initial conditions and demonstrates the coexistence of multiple attractors under identical parameter settings, underscoring the multistable nature of the system. Furthermore, we explored the concept of offset boosting to shift attractor positions in phase space without altering the underlying chaotic behavior. This feature provides a potential pathway for developing practical control mechanisms in real-world supply chain systems, especially those operating in uncertain or volatile environments. Overall, the proposed model contributes to the growing body of research on chaotic supply chain dynamics by offering a more flexible and controllable framework.

## Data Availability

The data that support the findings of this study are available from the corresponding author on reasonable request.
